# Predictive Value of Routine Imaging for the Diagnosis of Pathologic Complete Response After Neoadjuvant Chemotherapy in Breast Cancer

**DOI:** 10.1245/s10434-026-19670-6

**Published:** 2026-05-04

**Authors:** Maria Müller, Jörg Heil, André Pfob, Lie Cai, Eva Reisig, Riku Togawa, André Hennigs, Fabian Riedel, Anne Stieber, Manuel Feisst, Juliane Nees, Andreas Schneeweiss, Peter Sinn, Oliver Zivanovic, Michael Golatta, Benedikt Schäfgen

**Affiliations:** 1 Breast Unit, Department of Obstetrics and Gynecology, Heidelberg University Hospital, Heidelberg, Germany; 2 Breast Unit, Sankt Elisabeth Hospital, Heidelberg, Germany; 3 Department of Diagnostic and Interventional Radiology, Heidelberg University Hospital, Heidelberg, Germany; 4 Institute of Medical Biometry (IMBI), Heidelberg University, Heidelberg, Germany; 5 National Center for Tumor Diseases, University Hospital and German Cancer Research Center Heidelberg, Heidelberg, Germany; 6 Institute of Pathology, University of Heidelberg, Heidelberg, Germany; 7 Department of Obstetrics and Gynecology, Münster University Hospital, Münster, Germany

**Keywords:** Neoadjuvant chemotherapy, Routine imaging, Prediction, Pathological response, Mammography, Ultrasound, Breast cancer

## Abstract

**Background:**

After neoadjuvant chemotherapy (NACT) of breast cancer, an accurate response assessment is essential for the de-escalation of surgical therapy. This study evaluated whether breast imaging procedures can reliably predict a pathologic complete response (pCR).

**Methods:**

This retrospective, monocenter study included 388 cases of invasive breast cancer treated by NACT. Most of the patients received mammography (MG) and ultrasound (US). In some cases, magnetic resonance imaging (MRI) was performed for special indications. The absence of residual tumor on imaging was defined as clinical complete response (cCR). Minimal signs of residual tumor were categorized as near-cCR. The primary endpoints were negative predictive values (NPVs) and false-negative rates (FNRs) of all imaging methods, combined and separately, for the whole cohort and subgroups of triple-negative (TN), human epidermal growth factor 2-positive (HER2+), and hormone receptor-positive (HR+)/HER2– breast cancer.

**Results:**

The overall pCR rate was 40.7 %, with most pCR cases having triple-negative breast cancer (TNBC) (*n* = 85, 57.8 %). The highest NPV (71.6 %) and the lowest FNR (11.0 %) in the overall cohort were achieved by the combination of ultrasound and mammography. In the TNBC subgroup, NPVs ranged from 78.1 % to 83.7 %, compared with lower NPVs in the HR+ subgroup (34.2–50.0 %). If near-cCR cases were defined together with cCR as a “negative” test result, the predictive accuracy of imaging decreased in the whole cohort (FNR, 34.8 %; NPV, 56.1 %).

**Conclusion:**

Standard imaging is insufficient to predict pCR reliably in an unselected cohort. Strict patient selection and additional diagnostics are necessary for safe confirmation of pCR.

Neoadjuvant chemotherapy within a multimodal treatment regimen is considered the standard of care for patients with high-risk breast cancer. Indications include locally advanced, unresectable, or inflammatory breast cancer; triple-negative (TNBC) or HER2+ breast cancer; and tumors with high histologic grading or proliferation index (Ki-67).^1–3^ The neoadjuvant treatment allows for a higher rate of breast-conserving surgery, in vivo sensitivity testing, and prognostic assessment.^1,2^

In recent years, the number of women who have received neoadjuvant chemotherapy as part of their breast cancer treatment has increased substantially.^2,4^ Depending on the biologic subtype, 17 % to 65 % of the patients receive neoadjuvant treatment.^4,5^ This often leads to a significant reduction in tumor size or even complete disappearance of the primary tumor. The proportion of patients with a pathologic complete response (pCR) after neoadjuvant chemotherapy has increased substantially in recent years.^5^ Overall, pCR rates of approximately 20 % have been reported,^6–9^ with even higher rates depending on the biologic subgroup, up to 65 % among TNBC patients.^5–9^

Yet even if the primary tumor completely disappears after neoadjuvant chemotherapy, subsequent surgery still is essential for diagnostic purposes.^10^ To date, the only way to adequately assess tumor response and diagnose pCR with sufficient certainty is by performing histopathologic analysis of a surgically resected specimen. From the point of view of patients and the health care system, it would be desirable to develop methods that can reliably predict the diagnosis of pCR without surgery. This is an essential step toward safe selective omission of surgery for exceptional responders after NACT.

Several studies have evaluated the predictive power of imaging (magnetic resonance imaging [MRI], mammography, ultrasound) to assess remission, but the results vary widely, in part due to the lack of standardized definitions for clinical complete response (cCR).^11–14^ There is a consensus to date that, imaging alone is not reliable enough to accurately predict pCR.^11–23^ A review summarized the predictive values reported across various studies.^15^ According to this review, the negative predictive values (NPVs) ranged from 33 % to 91 % for ultrasound and from 30 % to 91 % for mammography.^15^ Yet closer examination showed that these studies often used differing definitions for predictive values. Whereas some studies used the NPV to assess the ability to predict a pCR,^11,13,16,19,20^ other studies defined the positive predictive value (PPV) as the main metric for predicting pCR.^12,14,18,21,22^ In such cases, the NPV instead reflects the predictive value for identifying residual disease (i.e., non-pCR). This distinction is important for interpretation of the results from different studies. After adjustment for this difference in definition, the ability to correctly predict a pCR ranges between 20 % and 69 % for ultrasound^11–14,16–19^ and between 30 % and 54 % for mammography.^13,14,16–18^

Although MRI had the highest predictive power across studies, with the ability to correctly predict a pCR ranging from 44 % to 83 %,^11–14,16,20–22^ MRI is used only infrequently in routine clinical practice compared with mammography and ultrasound. Current European Society of Breast Cancer Specialists (EUSOMA) quality indicators recommend functional imaging methods such as breast MRI or contrast-enhanced mammography (CEM) before and after neoadjuvant therapy to optimize response assessment.^24^ According to previous EUSOMA quality indicators, MRI was recommended before and after neoadjuvant systemic therapy at a minimum standard of 60 %, with a target of 90 % as of 2017.^25^ The 2024 update recommends either breast MRI or CEM at a minimal standard of 70 % and a target of 90 %.^24^ Similarly, international guidelines from other countries recognize MRI as an important tool for response assessment after neoadjuvant therapy.^26–28^ However, access to MRI and CEM remains limited in many health care systems, and the German S3 guideline restricts MRI to selected cases that cannot be clarified with sufficient certainty by conventional diagnostics (mammography, ultrasound) and percutaneous biopsy.^10^ Consequently, conventional imaging methods continue to play a central role in daily clinical practice. Our study therefore specifically addressed this real-world setting by evaluating the diagnostic accuracy of routine imaging (ultrasound and mammography) for predicting pCR after neoadjuvant chemotherapy in breast cancer patients.

## Methods

### Study Design and Patients

**Fig. 1:**
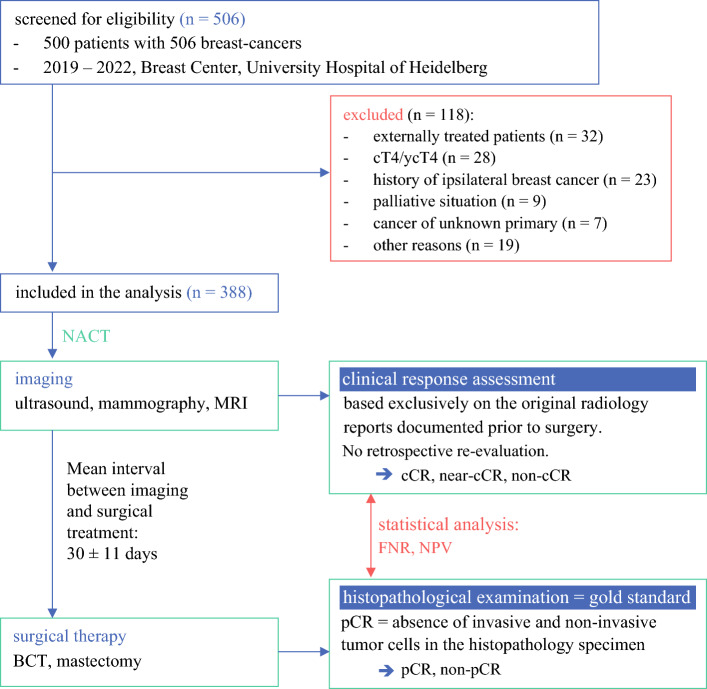
Study design

In this retrospective monocentric exploratory study, the data of 500 patients with 506 breast cancers treated at the Breast Center of the University Hospital of Heidelberg between 2019 and 2022 were screened for eligibility. Adult patients with breast cancer were screened if they had received neoadjuvant chemotherapy followed by surgical treatment. The exclusion criteria for the study ruled out externally treated patients (*n* = 32), clinical stage cT4 or ycT4 breast cancer (*n* = 28), history of ipsilateral breast cancer (*n* = 23), palliative situation and therefore no surgical therapy (*n* = 9), and cancer of unknown primary (*n* = 7). An additional 19 patients were excluded from the study for other reasons, such as loss to follow-up evaluation, patient refusal of neoadjuvant chemotherapy, and incomplete imaging findings. Finally, a total of 388 cases were included in the analysis (Fig. 1). Because this was a retrospective study based on existing institutional data, information on race and ethnicity was not routinely recorded and therefore was not available for analysis.

### Imaging: Implementation and Technique

All patients received neoadjuvant chemotherapy according to the standard protocols of our department, which are based on national guidelines.^10^ As per clinical routine, breast imaging was performed for these patients after completion of neoadjuvant chemotherapy but before surgical therapy. According to the German guidelines,^10^ ultrasound (*n* = 387) and mammography (*n* = 380) were performed regularly, whereas MRI (*n* = 39) was performed only for specific indications (e.g., multifocality/multicentricity). All imaging examinations were performed as part of routine clinical practice.

### Imaging: Evaluation

Imaging classifications were based exclusively on the original radiology reports documented before surgery. No retrospective re-evaluation or re-interpretation was conducted for the purpose of this analysis.

Because there is no standardized definition of clinical complete response (cCR) across the existing studies evaluating imaging after neoadjuvant chemotherapy, response categories were predefined for this analysis. Residual tumor findings detectable on imaging were classified as non-cCR (positive result), whereas complete absence of suspicious imaging findings was classified as cCR (negative result).

To account for equivocal post-neoadjuvant imaging findings frequently encountered in routine clinical reports, an additional “near-cCR” category was defined. The near-cCR category included cases in which radiology reports described the absence of a clearly delineable tumor lesion but documented minimal residual radiologic alterations at the former tumor site, such as scar-like changes, architectural distortion, faint shadowing, or residual microcalcifications.

In the primary analysis, near-cCR cases were classified as non-cCR, reflecting a strict definition of cCR consistent with routine clinical decision-making. In a secondary, exploratory analysis, near-cCR cases were alternatively added to cCR as a “negative” test result to assess the impact of equivocal imaging findings on predictive accuracy. Importantly, the near-cCR category does not represent a clinical decision category but was used exclusively for analytical purposes to assess the robustness of imaging-based response assessment.

Cases with imaging findings not clearly described in the radiology reports, which therefore could not be retrospectively categorized into one of the defined remission groups, were labeled as ycTX and considered unclear.

### Histopathologic Examination

To assess the predictive power of routine imaging for the diagnosis of pCR, the histopathologic findings of the surgical resection were used as the gold standard. Pathology was performed by experienced breast pathologists as part of the clinical routine. The definition of pCR differs between studies. In terms of prognosis, pathologic complete remission, defined as the absence of invasive and non-invasive tumor cells in the breast and lymph nodes, is the best way to discriminate between patients with good and poor outcomes.^29^ Accordingly, we defined pCR as the absence of invasive and non-invasive tumor cells in the histopathology specimen. Nodal status was not taken into account in this study.

### Statistical Analysis

The false-negative rate (FNR) was calculated as the number of patients with a false-negative result (cCR [or cCR and near-cCR]) on imaging but non-pCR on histopathology divided by the total number of patients with non-pCR on histopathology. The NPV was calculated as the number of patients with a true negative imaging result (both cCR [or cCR and near-cCR] and pCR) divided by the number of all the patients with a negative imaging result (cCR [or cCR and near-cCR]). The NPV indicates how likely it is that a patient with a clinical complete remission will actually have a pathologic complete remission. Both NPV and FNR were calculated for the different imaging methods and the different tumor biology subgroups. The 95 % confidence intervals (CIs) were calculated using the asymptotic normal distribution method based on binomial distribution. Statistical analysis was performed using IBM SPSS Statistics for Windows, version 28, 2024 (IBM Corp., Armonk, N.Y., USA).

## Results

### Patients and Tumor Characteristics

Data from 388 of 506 breast cancer cases were included in the analysis, with 50 % (*n* = 194) of the cancers located on the left and 50 % (*n* = 194) on the right. The mean age of the patients at the time of diagnosis was 49.4 ± 11.9 years (range, 21–78 years). At the time of diagnosis, 47 % (*n* = 183) of the patients were premenopausal and 43 % (*n*=168) were postmenopausal. The greatest portion of tumors were non-specific invasive ductal carcinomas, stage cT2, triple-negative tumors (Table 1).

**Table 1:** Patient characteristics

	*n*	%
*Histologic type*		
NST	359	92.5
Invasive lobular	12	3.1
Other	17	4.4
*Subtype*		
TNBC	147	37.9
HER2–/HR+	136	35.1
HER2+	105	27.1
*cT stage*		
cT1a	0	0.0
cT1b	9	2.3
cT1c	105	27.1
cT2	236	60.8
cT3	38	9.8
*Grading*		
G1	3	0.8
G2	145	37.4
G3	238	61.3
GX	2	0.6

NST, no special type; TNBC, triple-negative breast cancer; HER2, human epidermal growth factor 2; HR, hormone receptor

In 379 cases, both ultrasound and mammography were performed, and 37 patients underwent ultrasound, mammography, and MRI. The mean interval between imaging and surgical treatment was 30 ± 11 days.

The majority of the patients were treated with either breast-conserving therapy (BCT) (*n* = 222) or mastectomy (*n* = 160). A small subset (*n* = 6) received BCT followed by mastectomy. The rate of pCR varied substantially by tumor biology subgroup (Table 2).

**Table 2:** Imaging and surgery details and assessment

	(a) Imaging assessment					
	US (*n* = 387)		Mammography (*n* = 380)		MRI (*n* = 39)	
	*n*	%	*n*	%	*n*	%
cCR	150	38.8	129	33.9	9	23.1
Near-cCR	67	17.3	68	17.9	22	56.4
Non-cCR	169	43.7	119	31.3	8	20.5
Unclear	1	0.3	64	16.8	0	0.0

(b) Histopathologic assessment		
pCR-rate	*n*	%
All (*n* = 388)	158	40.7
TNBC (*n* = 147)	85	57.8
HER2–/HR+ (*n* = 136)	21	15.4
HER2+ (*n* = 105)	52	49.5

ypT stage	*n*	%
ypT0 (pCR)	158	40.7
ypTis	36	9.3
ypT1mic	2	0.5
ypT1a	34	8.8
ypT1b	46	11.9
ypT1c	55	14.2
ypT2	46	11.9
ypT3	11	2.8

US, ultrasound; MRI, magnetic resonance imaging; cCR, clinical complete response; pCR, pathologic complete response; TNBC, triple-negative breast cancer; HER2, human epidermal growth factor 2; HR, hormone receptor

### Accuracy of Ultrasound and Mammography for the Whole Cohort

In the whole cohort, the FNR was 26.6 % for ultrasound alone, 19.7 % for mammography alone, and 11.0 % for ultrasound and mammography combined (false-positive result for both ultrasound and mammography). The NPV was 59.3 % for ultrasound alone, 65.1 % for mammography alone, and 71.6 % for ultrasound and mammography combined (true-negative result for both ultrasound and mammography).

### Accuracy of Ultrasound and Mammography by Receptor Status

The FNR for combined imaging ranged from 9.7 % (HR+ cancers) to 13.2 % (HER2+ cancers). Differences between tumor biology subgroups were especially evident when NPV was examined. In the triple-negative group, the NPV of combined ultrasound and mammography was 83.7 %, whereas HR+/HER2– carcinomas with a similar FNR had an NPV of only 50.0 %.

### Role of MRI

Our study focused on the predictive power of routine imaging consisting of ultrasound, mammography, or both. In 39 cases, MRI was performed in addition to ultrasound or mammography because of a clinical indication, and in 37 of these cases, all three imaging methods (ultrasound, mammography, and MRI) were combined. None of the 25 cases with residual disease were misdiagnosed as cCR. Subgroup analysis was not performed due to the small number of cases. For detailed results on the FNR and NPV, see Table 3.

**Table 3:** FNR and NPV (cCR group)

Total (*n* = 388)	*n*	FNR (%)	95 % CI	NPV (%)	95 % CI
US	387	26.6	20.9–32.4 %	59.3	51.5–67.2 %
Mammography	380	19.7	14.0–25.5 %	65.1	56.9–73.3 %
MRI	39	8.0	0.0–18.6 %	77.8	50.6–100.0 %
US + mammography	379	11.0	6.9–15.1 %	71.6	62.2–81.0 %
US + mammography + MRI	37	0.0	–	100.0	–
*TNBC (n = 147)*					
US	147	19.4	9.5–29.2 %	81.3	71.7–90.8 %
Mammography	143	23.0	11.2–34.7 %	78.1	68.0–88.3 %
MRI	10	0.0	–	100.0	–
US + mammography	143	11.5	3.4–19.5 %	83.7	72.7–94.7 %
US + mammography + MRI	10	0.0	–	100.0	–
*HR+/HER2– (n = 136)*					
US	135	23.9	15.9–31.5 %	34.2	19.6–48.7 %
Mammography	135	18.4	10.6–26.2 %	38.2	21.9–54.6 %
MRI	16	7.2	0.0–20.6 %	0.0	–
US + mammography	134	9.7	4.2–15.3 %	50.0	29.1–70.9 %
US + mammography + MRI	16	0.0	–	–	–
*HER2+ (n = 105)*					
US	105	41.5	28.2–54.8 %	51.1	36.5–65.7 %
Mammography	102	18.9	6.9–30.8 %	67.7	51.3–84.2 %
MRI	13	14.3	0.0–40.2 %	75.0	32.6–100.0 %
US + mammography	102	13.2	4.0–22.4 %	69.6	50.8–88.4 %
US + mammography + MRI	11	0.0	–	100.0	–

FNR, false-negative rate; NPV, negative predictive value; cCR, clinical complete response; CI confidence interval; US, ultrasound; MRI, magnetic resonance imaging; TNBC, triple-negative breast cancer; HR, hormone receptor; HER2, human epidermal growth factor 2

### Analysis of False-Negative Cases

In the combined imaging of ultrasound and mammography, 25 of 227 non-pCR cases were missed (FNR, 11.0 %). The size of the invasive residual tumor was smaller than 1 cm in 18 of the 25 cases, including 5 cases with ypTis. The residual tumor was 1 to 2 cm in five cases and larger than 2 cm in two cases.

### Subgroup Analysis of the Near-cCR Category (Secondary Analysis)

This analysis was exploratory and not intended to reflect routine clinical practice. The results described earlier refer to the strict cCR definition, in which only cCR cases were considered negative. Using the broad definition considering near-cCR together with cCR as a “negative” test result, the predictive accuracy of imaging was reduced in all subgroups. The FNR increased by 16.6 percentage points to 43.2 % for ultrasound, by 19.8 percentage points to 39.7 % for mammography, and by 23.8 percentage points to 34.8 % for combined imaging. Correspondingly, the NPV decreased for each imaging method (e.g., from 71.6 % [strict cCR definition] to 56.1 % [cCR + near-cCR] for combined imaging in the entire cohort). For detailed results for the cCR + near-cCR group, see Table 4.

**Table 4:** FNR and NPV (cCR/near-cCR group)

Total (*n* = 388)		FNR (%)	95 % CI	NPV (%)	95 % CI
US	387	43.2	36.8–49.6 %	54.4	47.8–61.0 %
Mammography	380	39.5	32.4–46.5 %	54.3	47.4–61.3 %
MRI	39	68.0	49.7–86.3 %	45.2	27.6–62.7 %
US + mammography	379	34.8	28.5–41.1 %	56.1	48.9–63.4 %
US + mammography + MRI	37	40.0	20.8–59.2 %	52.4	31.0–73.7 %
*TNBC (n = 147)*					
US	147	38.7	26.6–50.8 %	73.0	63.8–82.3 %
Mammography	143	39.3	25.7–53.0 %	71.8	62.2–81.3 %
MRI	10	25.0	0.0–67.4 %	85.7	59.8–100.0 %
US + mammography	143	31.2	19.4–42.9 %	74.3	64.4–84.3 %
US + mammography + MRI	10	0.0	–	100.0	–
*HR+/HER2– (n = 136)*					
US	135	40.4	31.3–49.4 %	28.2	17.1–39.1 %
Mammography	135	34.2	24.7–43.8 %	27.8	15.8–39.7 %
MRI	16	64.3	39.2–89.4 %	18.2	0.0–41.0 %
US + mammography	134	30.1	21.5–38.7 %	32.0	19.1–44.9 %
US + mammography + MRI	16	28.6	4.9–52.2 %	20.0	0.0–55.1 %
*HER2+ (n = 105)*					
US	105	54.7	41.3–68.1 %	54.7	42.5–66.9 %
Mammography	102	50.9	35.6–66.3 %	53.5	40.6–66.3 %
MRI	13	100.0	–	46.2	19.1–73.3 %
US + mammography	102	49.1	35.5–62.6 %	53.6	40.5–66.6 %
US + mammography + MRI	11	85.7	59.8–100.0 %	40.0	9.6–70.4 %

FNR, false-negative rate; NPV, negative predictive value; cCR, clinical complete response; CI confidence interval; US, ultrasound; MRI, magnetic resonance imaging; TNBC, triple-negative breast cancer; HR, hormone receptor; HER2, human epidermal growth factor 2

## Discussion

Diagnostic procedures with a low FNR and a high NPV are essential to predict pCR after NACT and identify candidates for a potential de-escalation of surgical treatment. On the other hand, it is crucial to reliably detect any residual disease after NACT to avoid undertreatment.

Our results showed that conventional imaging alone does not predict pCR with sufficient certainty, consistent with previous studies.^11–22^ The highest NPV in our overall cohort was 71.6 %, achieved by the combination of ultrasound and mammography. This combination also had the lowest FNR (11.0 %).

In our study, MRI was used for only a small number of patients because it is used only for specific indications according to German guidelines. Our analysis showed that MRI is superior to both ultrasound and mammography, consistent with the results of previous studies.^11–13,16^ Interestingly, the combination of ultrasound, mammography, and MRI achieved an FNR of 0 % and an NPV of 100 % using the strict cCR definition. However, due to the small number of cases, these results should be interpreted with caution, and larger sample sizes are needed to verify these findings.

Current EUSOMA quality indicators recommend the use of functional imaging methods, such as breast MRI or contrast-enhanced mammography, before and after neoadjuvant systemic therapy to optimize response assessment.^24^ Our findings support this recommendation by demonstrating the limited ability of conventional imaging methods to reliably predict pCR, particularly in an unselected cohort. However, the implementation of functional imaging remains heterogeneous across health care systems, and access to these methods remains limited in many clinical settings. In this context, the current study provides important real-world data on the diagnostic performance of routinely used imaging methods and highlights the need for additional diagnostic strategies beyond conventional imaging when surgical de-escalation after neoadjuvant therapy is considered.

As observed in other studies,^12,13,19–22^ the NPV is strongly dependent on tumor biology. In our study, NPVs for triple-negative carcinomas ranged from 78.1 % to 83.7 %, but they were significantly lower for HR+ carcinomas (34.2–50.0 %). These differences were due to the different pCR rates of the tumor subgroups, which strongly influenced the predictive values. The predictive values should therefore be interpreted with caution and always in the context of the false-negative rate.

Although the Response Evaluation Criteria in Solid Tumors (RECIST) criteria provide standardized guidelines for assessing response on imaging, they refer to solid tumors in general and are not specifically tailored to the characteristics of breast tumors.^30^ The introduction of the near-cCR category was intended to address the frequent occurrence of equivocal post-NACT imaging findings in routine radiology reports, in which imaging does not allow a clear classification as cCR despite the absence of unequivocal residual tumor. As demonstrated by the marked decline in diagnostic accuracy when these cases were included, our results underscore the limitations of conventional imaging and support the use of strict response definitions in clinical decision-making. At the same time, the separate analysis of near-cCR cases highlights the need for more refined classification systems and additional diagnostic strategies to improve the interpretation of post-NACT imaging findings.

The strength of our study was the large sample size with a total of 388 cases included. To our knowledge, no similarly large study has investigated the predictive power of routine imaging for the diagnosis of pCR. The evaluation of routinely used imaging methods leads to a high degree of practical relevance and applicability of the results to everyday clinical practice. The predictive power of combined imaging methods compared with single methods has been evaluated in only a few studies.^12,17^ Clearly defined response categories help to ensure the reproducibility of our results. The introduction of the near-CCR category for all three imaging methods allows borderline cases to be identified and specifically analyzed. This contributes to the refinement of diagnostic methods and provides a basis for further development of algorithms and prediction models.

The main limitation of our study was its retrospective design because all data were collected from routine clinical findings. This may have biased the results because no standardized imaging protocols were used, and some findings had to be classified as equivocal due to inaccurate information. Furthermore, the small number of cases does not allow reliable statements to be made about the predictive value of MRI. Also, the study was monocentric at a large European university hospital, so the results may not be representative of other settings.

In addition to optimizing existing imaging techniques, innovative diagnostic approaches are becoming increasingly important. Minimally invasive procedures such as vacuum-assisted biopsy (VAB) are a promising complement to imaging. By removing tissue from the area previously harboring the tumor after neoadjuvant chemotherapy, pCR can be assessed with greater accuracy. Studies such as RESPONDER have shown that the use of VAB significantly increases diagnostic confidence.^31–34^ Although VAB alone did not meet the target of an FNR rate below 10 %, an FNR of 6.2 % was achieved in combination with imaging.^32^ The use of intelligent algorithms opens up new opportunities to further improve the predictive value of imaging. Machine-learning models that integrate large datasets of imaging, clinical parameters, and histopathologic results have shown promising results in accurately differentiating between pCR and non-pCR.^35,36^ These AI-based approaches might not only improve diagnostic accuracy in the future, but also support personalized treatment planning. In combination with advanced imaging methods, these approaches have the potential to avoid unnecessary surgical intervention for patients with pCR.

## Conclusion

In conclusion, consistent with previous studies,^10–21^ our results show that conventional imaging alone does not predict pCR with sufficient certainty.
